# Low estimated glomerular filtration rate and high body mass index are risk factors for acute kidney injury in systemic lupus erythematosus patients after cardiac surgery

**DOI:** 10.3389/fcvm.2024.1387612

**Published:** 2024-06-07

**Authors:** Xue Zhang, Chunrong Wang, Yajie Tian, Yuelun Zhang, Qi Miao, Di Wu, Chunhua Yu

**Affiliations:** ^1^ Department of Anesthesiology, Chinese Academy of Medical Sciences, Peking Union Medical College Hospital, Beijing, China; ^2^ Medical Research Center, Chinese Academy of Medical Sciences, Peking Union Medical College Hospital, Beijing, China; ^3^ Department of Cardiac Surgery, Chinese Academy of Medical Sciences, Peking Union Medical College Hospital, Beijing, China; ^4^ Department of Rheumatology, Chinese Academy of Medical Sciences, Peking Union Medical College Hospital, Beijing, China

**Keywords:** systemic lupus erythematosus, acute kidney injury, cardiac surgery, estimated glomerular filtration rate, body mass index, risk factor

## Abstract

**Objective:**

The occurrence of acute kidney injury (AKI) is common following cardiac surgery, especially among patients characterized with systemic lupus erythematosus (SLE), but studies on this clinical scenario have been limited by the rarity of SLE. We aimed to explore the risk predictors and outcomes with regards to postoperative AKI among cardiac-surgical patients concomitant with SLE.

**Methods:**

This was a single-center retrospective study performed in a tertiary hospital. Adult patients diagnosed with SLE who underwent cardiac surgery within the last 22 years were enrolled. Essential variables, including patient-, surgery- and anesthesia-related information, were collected from the medical record system. The definition of AKI was derived from the Kidney Disease: Improving Global Outcomes (KDIGO) criteria. Risk predictors suspected to be linked with post-surgical AKI were calculated using the univariable and multivariable analyses.

**Results:**

Of all 59 SLE patients undergoing cardiac surgery, 57 were ultimately enrolled into the analysis. AKI occurred in 29 patients (50.9%), who had significantly longer extubation time (median difference 1.0 day, *P* < 0.001), ICU length of stay (median difference 2.0 days, *P* = 0.001), postoperative length of stay (median difference 5.0 days, *P* = 0.026), and more postoperative major complications (odds ratio 10.29, *P* = 0.025) than the others. Preoperative estimated glomerular filtration rate (eGFR) < 60 ml/min/1.73 m^2^ (odds ratio 5.31, *P* = 0.021) and body mass index (BMI) ≥ 24 kg/m^2^ (odds ratio 4.32, *P* = 0.043) were the only two factors in the multivariable analysis that were significantly correlated with the development of postoperative AKI in patients with SLE after cardiac surgery.

**Conclusion:**

AKI in SLE patients after cardiac surgery is common and requires scrutiny, especially in overweight patients with moderate to severe preoperative renal dysfunction.

## 1 Introduction

Systemic lupus erythematosus (SLE) is an autoimmune disease that has deleterious effects on multiple systems, mainly the heart, kidneys, lungs and nervous system (1). As therapeutic approaches evolve, overall mortality secondary to SLE complications has promisingly decreased, but mortality hazard from cardiovascular burden has remained steady in SLE patients (2). Patients with long-term SLE owned a three- to fourfold increased hazard of cardiovascular events and cardiovascular-related mortality in comparison with those without SLE, despite no disparity in the distribution of traditional cardiovascular risk factors (3). Moreover, most components of the cardiovascular system, such as large vessels, valves, coronary arteries and the pericardium, can be involved in active SLE (4, 5). Cardiac surgeries are sometimes indicated when patients have severe clinical manifestations. Since cardiac surgery is not performed routinely, few studies on the risk factors for poor outcomes after cardiac surgery in SLE patients have been done, this information being restricted to case reports (6–11).

Acute kidney injury (AKI) following cardiac surgery is a causative factor of extended recovery time; as well, it increased health care costs and contributed strongest to long-term mortality (12–14). Our previous study demonstrated that in comparison with patients concomitant with vasculitis, those with SLE were more prone to be vulnerable to AKI after on-pump cardiac surgery (15). The present study aimed to further clarify risk factors and consequent outcomes regarding postoperative AKI among the patient cohort of SLE after cardiac procedures.

## 2 Materials and methods

### 2.1 Patient enrollment

The Institutional Review Board of Peking Union Medical College Hospital approved this study before its initiation (S-K2063, November 7, 2023). Owning to its retrospective nature, the written informed consent was accordingly waived, and the data were anonymized. We searched the hospital medical record system to find information involving all adult patients concomitant with SLE who underwent open-heart cardiac surgery between March 2002 and October 2023. The exclusion criteria were ① death within seven days postoperatively and ② need for preoperative renal replacement therapy.

### 2.2 Data collection

Essential variables were collected from our medical record system by research group members who had received uniform training on data extraction before starting this research. Preoperative characteristics, including sex, age, height, weight, heart function class, ever/current smoking and alcohol usage, comorbidities, course of SLE, disease activity, treatment, and laboratory test results, were extracted. Surgical information (surgery type, duration, cardiopulmonary bypass duration and aortic cross-clamping duration) and anesthesia-related information (American Society of Anesthesiologists physical status, intraoperative fluid, blood product and vasoactive drug usage) were also collected. The calculation of estimated glomerular filtration rate (eGFR) was achieved via the Chronic Kidney Disease Epidemiology Collaboration equation (16).

The definition of AKI was derived from the Kidney Disease: Improving Global Outcomes (KDIGO) criteria. It possessed higher sensitivity in detecting AKI compared with either the risk of renal dysfunction, injury to the kidney, failure of kidney function, loss of kidney function, the end-stage kidney disease (RIFLE) criteria or the Acute Kidney Injury Network criteria (17). AKI was ascertained as one of the following occurred: (1) an increase in serum creatinine ≥0.3 mg/dl (26.5 μmol/l) within 48 h, (2) an increase in serum creatinine to ≥1.5 times the baseline within 7 days postoperatively, or (3) urine output <0.5 ml/kg/h for 6 h (18, 19).

As for the outcome data, major complications were classified as a composite outcome before discharge that included the need for extracorporeal membrane oxygenation, the use of an intra-aortic balloon pump, paravalvular leakage, valve detachment, infection, reoperation, and cerebrovascular accident. In addition, duration of mechanical ventilation, and length of stay (LOS) in intensive care unit (ICU) and total postoperative in-hospital LOS were also recorded.

### 2.3 Statistical analysis

Continuous variables distributed normally were expressed as the mean ± standard deviation, while those distributed nonnormally as median (quartiles). The comparisons between continuous data were achieved using the independent *t*-test or Mann‒Whitney *U*-test. Categorical variables were presented as number (percentage) and compared using the chi-square (*χ*^2^) test or Fisher's exact test when appropriate. Continuous data, including body mass index, eGFR, erythrocyte sedimentation rate, and C-reactive protein, were all divided into categorical variables on the basis of cutoffs routinely used in the clinical scenarios. The univariable analysis was employed to detect out potential factors contributed to the development of post-surgical AKI. Then the multivariable analysis, incorporating variables of *P* < 0.1 on univariable analysis or with significant clinical values, was ultimately performed. A logistic regression model was built by the enter method to screen for correlated factors. Statistical significance was considered if a *P*-value < 0.05. All statistical analyses involving in this study were performed via SPSS (IBM SPSS statistics Version 26, Chicago, IL, USA).

## 3 Results

### 3.1 Patient characteristics

A total of 59 patients with SLE underwent cardiac surgery through median sternotomy at our institution over the period of 22 years. Two patients died four days after surgery and were excluded, leaving 57 patients for analysis. Twenty-nine (50.9%) of the patients developed AKI were assigned to the AKI group, while the remaining 28 patients constituted the non-AKI group.

Tables 1 and 2 displayed patient baseline information. Patients in the AKI group had significantly lower preoperative hemoglobin (102.6 ± 21.3 g/L vs. 113.8 ± 16.6 g/L, mean difference of −11.2 g/L, *P* = 0.031) and eGFR (67.3 ± 44.9 vs. 93.8 ± 36.7 ml/min/1.73 m^2^, mean difference of −26.56 ml/min/1.73 m^2^, *P* = 0.018) than patients in the non-AKI group. There were more patients with moderate to severe preoperative renal dysfunction (eGFR < 60 ml/min/1.73 m^2^) in the AKI group than in the non-AKI group (17 vs. 4, odds ratio 8.50; *P* = 0.001). There were more overweight patients (BMI ≥ 24 kg/m^2^) in the AKI group than in the control group, while no significant difference was revealed (12 vs. 6, odds ratio 2.93, *P* = 0.069).

**Table 1:** Demographic and preoperative baseline characteristics of patients with and without postoperative AKI.

Preoperative characteristics	All patients (mean ± SD/N, %/median, IQR)	AKI group (mean ± SD/N, %/median, IQR)	Non-AKI group (mean ± SD/N, %/median, IQR)	Odds ratio/mean difference/median difference (95% CI)	*P*
Age (years old)	41.0 (26.5)	50.0 (27.5)	41.0 (21.3)	6.00 (−2.00 to 14.00)	0.140
Sex					
Male	9 (15.8)	5 (17.2)	4 (14.3)	1.25 (0.30 to 5.24)	1.000
Female	48 (84.2)	24 (82.8)	24 (85.7)		
Elective/Emergency					
Elective	53 (93.0)	26 (89.7)	27 (96.4)	0.32 (0.03 to 3.29)	0.611
Emergency	4 (7.0)	3 (10.3)	1 (3.6)		
Height (cm)	165.0 ± 6.3	165.2 ± 6.4	164.9 ± 8.3	0.29 (−3.14 to 3.72)	0.865
Weight (kg)	60.6 ± 12.6	62.5 ± 13.9	58.6 ± 11.0	3.96 (−2.69 to 10.61)	0.237
BMI (kg/cm^2^)	22.3 ± 4.2	23.2 ± 5.0	21.4 ± 3.3	1.14 (−0.51 to 4.07)	0.124
BMI ≥ 24 kg/m^2^	18 (32.7)	12 (44.4)	6 (21.4)	2.93 (0.90 to 9.54)	0.069
Smoking history	7 (12.3)	4 (13.8)	3 (10.7)	1.33 (0.27 to 6.58)	1.000
Drinking history	3 (5.3)	2 (6.9)	1 (3.6)	2.00 (0.17 to 23.4)	1.000
Comorbidities					
Hypertension	23 (40.4)	12 (41.4)	11 (39.3)	1.09 (0.38 to 3.15)	0.872
Diabetes	4 (7.0)	3 (10.3)	1 (3.6)	3.12 (0.30 to 31.90)	0.611
CVD	12 (21.1)	7 (24.1)	5 (17.9)	1.46 (0.40 to 5.31)	0.561
Course of SLE (months)	72.0 (157.5)	84.0 (138.0)	48.0 (178.75)	16.00 (−22.00 to 69.00)	0.299
ASA physical status					
I-II	4 (7.0)	2 (6.9)	2 (7.1)	0.96 (0.13 to 7.35)	1.000
III-IV	53 (93.0)	27 (93.1)	26 (92.9)		
NYHA heart function					
I-II	23 (41.1)	9 (31.0)	14 (51.9)	0.42 (0.14 to 1.24)	0.174
III-IV	33 (58.9)	20 (69.0)	13 (48.1)		
White blood cells (×10^9^/l)	7.3 ± 5.6	7.4 ± 4.9	7.3 ± 6.3	0.10 (−2.91 to 3.11)	0.948
Percentage of neutrophils (%)	69.1 ± 13.1	71.5 ± 13.5	66.7 ± 12.4	4.75 (−2.12 to 11.62)	0.171
Hemoglobin (g/l)	108.1 ± 19.8	102.6 ± 21.3	113.8 ± 16.6	−11.20 (−21.31 to −1.09)	0.031
Platelet (×10^9^/l)	148.0 (107.0)	152.0 (94.0)	136.0 (130.3)	13.50 (−24.0 to 50.0)	0.539
Alanine aminotransferase (U/l)	17.0 (14.0)	18.0 (13.5)	16.0 (13.5)	−1.00 (−6.00 to 5.00)	0.761
Albumin (g/l)	36.0 (10.0)	36.0 (9.5)	36.5 (9.5)	−1.00 (−4.00 to 2.00)	0.512
eGFR (ml/min/1.73 m^2^)	80.3 ± 42.8	67.3 ± 44.9	93.8 ± 36.7	−26.56 (−48.30 to −4.82)	0.018
eGFR < 60 ml/min/1.73 m^2^	21 (36.8)	17 (58.6)	4 (14.3)	8.50 (2.34 to 30.91)	0.001
Prothrombin time (s)	11.6 (1.70)	11.8 (2.0)	11.5 (1.7)	0.40 (−0.20 to 1.00)	0.244
Activated partial thromboplastin time (s)	29.9 (7.0)	29.1 (8.4)	30.2 (6.1)	−0.40 (−3.20 to 2.70)	0.811
Fibrinogen (mg/dl)	2.8 ± 1.0	2.9 ± 1.0	2.6 ± 0.9	0.28 (−0.23 to 0.79)	0.277
Erythrocyte sedimentation rate (mm)	13.5 (24.3)	13.0 (17.0)	14.0 (31.0)	0.00 (−11.00 to 6.00)	0.972
C-reactive protein (mg/dl)	1.7 (9.1)	1.7 (8.0)	1.4 (12.5)	−0.11 (−2.51 to 1.26)	0.776

AKI, acute kidney injury; IQR, interquartile range; CI, confidence interval; BMI, body mass index; CVD, cerebrovascular disease; SLE, systemic lupus erythematosus; ASA, American Society of Anesthesiologists; NYHA, New York Heart Association; eGFR, estimated glomerular filtration rate.

**Table 2:** Preoperative chronic kidney disease stage in patients with and without postoperative AKI.

Chronic kidney disease stage	AKI group (*N*, %)	Non-AKI group (*N*, %)
Stage I	7 (24.1)	13 (46.4)
Stage II	5 (17.2)	11 (39.3)
Stage III	11 (37.9)	2 (7.1)
Stage IV	2 (6.9)	2 (7.1)
Stage V	4 (13.8)	0 (0.0)

AKI, acute kidney injury.

### 3.2 Surgery- and anesthesia-related information

Most of the patients in our study (*n* = 38, 66.7%) underwent valvular repair or replacement. Several underwent coronary artery bypass grafting (*n* = 10, 17.5%). Moreover, no significant differences were detected between the two groups with regards to surgery- or anesthesia-related parameters (Table 3).

**Table 3:** Comparison of surgery- and anesthesia-related data between patients with and without postoperative AKI.

Intraoperative characteristics	AKI group (*N*, %/median, IQR)	Non-AKI group (*N*, %/median, IQR)	Median difference (95% CI)	*P*
Type of surgery			–	0.683
Valvuloplasty/valve replacement	18 (62.1)	20 (71.4)		
Aortic root/ascending aorta replacement	2 (6.9)	0 (0.0)		
Coronary artery bypass grafting	5 (17.2)	5 (17.9)		
Pericardiectomy	1 (3.4)	1 (3.6)		
Combined surgery	3 (10.3)	2 (7.1)		
Surgery time (min)	300.0 (132.5)	292.5 (133.8)	20.00 (−30.00 to 65.00)	0.384
CPB time (min)	139.0 (72.3)	125.0 (66.5)	0.00 (−25.00 to 31.00)	0.970
ACx time (min)	80.5 (42.0)	83.0 (64.5)	−4.00 (−26.00 to 15.00)	0.585
Red blood cell transfusion (ml)	0.0 (600.0)	0.0 (400.0)	0.00 (0.00 to 0.00)	0.971
Plasma transfusion (ml)	400.0 (500.0)	400.0 (400.0)	0.00 (0.00 to 200.00)	0.476
Platelet transfusion (ml)	200.0 (300.0)	0.0 (200.0)	0.00 (0.00 to 200.00)	0.347
VIS before leaving the OR	13.5 (16.5)	7.0 (7.5)	4.50 (0.00 to 11.00)	0.128

AKI, acute kidney injury; IQR, interquartile range; CI, confidence interval; CPB, cardiopulmonary bypass; ACx, aortic cross-clamping; VIS, vasoactive-inotropic score; OR, operating room.

### 3.3 Correlated factors of postoperative AKI in SLE patients

In the multivariable logistic regression model, confounders with *P* < 0.1 on univariable analysis and those with clinical significance were adjusted for. The results showed that a preoperative eGFR < 60 ml/min/1.73 m^2^ (odds ratio 5.31, *P* = 0.021) and BMI ≥ 24 kg/m^2^ (odds ratio 4.32, *P* = 0.043) were independently correlated with postoperative AKI occurrence in patients with SLE (Table 4). Preoperative hemoglobin level was not identified as a risk predictor of AKI (odds ratio 0.97, *P* = 0.105).

**Table 4:** Multivariable analysis of potential factors correlated with postoperative acute kidney injury in patients with SLE.

Potentially correlated factors	Odds ratio (95% CI)	*P*
Preoperative hemoglobin (g/L)	0.97 (0.93 to 1.01)	0.105
BMI ≥ 24 kg/m^2^	4.32 (1.05 to 17.76)	0.043
eGFR < 60 ml/min/1.73 m^2^	5.31 (1.28 to 22.01)	0.021

SLE, systemic lupus erythematosus; CI, confidence interval; BMI, body mass index; eGFR, estimated glomerular filtration rate.

### 3.4 Outcome and treatment data

Forty-four patients (77.2%) were receiving glucocorticoids, and thirty-three (57.9%) were given antimalarial drug perioperatively, among whom 30 patients (52.6%) were taking both. As for outcomes, patients in the AKI group had significantly extended extubation time (3.0 vs. 1.0 days, median difference 1.0 day, *P* < 0.001), ICU LOS (4.0 vs. 2.0 days, median difference 2.0 days, *P* = 0.001) and postoperative LOS (16.0 vs. 10.5 days, median difference 5.0 days, *P* = 0.026). Eight patients in the AKI group had major postoperative complications, while only one patient in the non-AKI group had significant complications. The outcomes are detailed in Table 5. Medical treatment data was shown in Table 6.

**Table 5:** Outcomes of patients with or without postoperative AKI.

Outcome	AKI group (*N*, %/median, IQR)	Non-AKI group (*N*, %/median, IQR)	Median difference/odds ratio (95% CI)	*P*
Postoperative complications	8 (27.6)	1 (3.6)	10.29 (1.19 to 88.80)	0.025
Use of ECMO	1 (3.4)	0 (0.0)	1.04 (0.97 to 1.11)	1.000
Use of IABP	3 (10.3)	0 (0.0)	1.12 (0.99 to 1.26)	0.237
Paravalvular leakage	2 (6.9)	0 (0.0)	1.07 (0.97 to 1.19)	0.491
Valve detachment	0 (0.0)	0 (0.0)	–	–
Infection	1 (3.4)	0 (0.0)	1.04 (0.97 to 1.11)	1.000
Reoperation	1 (3.4)	1 (3.6)	0.96 (0.06 to 16.21)	1.000
Cerebrovascular accident	0 (0.0)	0 (0.0)	–	–
Extubation time (days)	3.0 (4.5)	1.0 (1.0)	1.00 (1.00 to 3.00)	<0.001
ICU LOS (days)	4.0 (5.5)	2.0 (2.8)	2.00 (1.00 to 4.00)	0.001
Postoperative LOS (days)	16.0 (21.0)	10.5 (15.8)	5.00 (1.00 to 11.00)	0.026
Total hospital LOS (days)	32.0 (33.0)	20.0 (25.3)	5.00 (−2.00 to 17.00)	0.253

AKI, acute kidney injury; IQR, interquartile range; OR, odds ratio; ECMO, extracorporeal membrane oxygenation; IABP, intra-aortic balloon pump; ICU, intensive care unit; LOS, length of stay.

**Table 6:** Medical treatment data of patients in AKI and non-AKI group.

Treatment	Total (*N*, %)	AKI group (*N*, %)	Non-AKI group (*N*, %)
Glucocorticoids	44 (77.2)	24 (82.8)	20 (71.4)
Hydroxychloroquine	22 (38.6)	10 (34.5)	12 (42.9)
Azathioprine	1 (1.8)	1 (3.4)	0 (0.0)
Tacrolimus	3 (5.3)	2 (6.9)	1 (3.6)
Cyclophosphamide	5 (8.8)	3 (10.3)	2 (7.1)
Leflunomide	1 (1.8)	0 (0.0)	1 (3.6)
Methotrexate	3 (5.3)	2 (6.9)	1 (3.6)
Mycophenolate mofetil	4 (7.0)	2 (6.9)	2 (7.1)
Unknown	3 (5.3)	2 (6.9)	1 (3.6)

AKI, acute kidney injury.

## 4 Discussion

With the improvement of anesthesia management (e.g., low dose of opioid facilitate enhanced recovery after surgery), surgical techniques (decreased time of surgery and cardio-pulmonary bypass) and implementation of various perioperative monitoring and supporting facilities (e.g., intraoperative transesophageal echocardiography and cerebral oxygen saturation monitor, high flow oxygen inhalation in ICU, ECMO support in critical patients), cardiac surgery has become safer in recent decades. However, cardiac surgery–associated AKI is still common for many reasons, such as renal hypoperfusion and reperfusion injury after ischemia, perioperative use of nephrotoxic drugs, metabolic and neurohormonal activation, as well as inflammation and oxidative stress (20). In relation to general population, cardiac involvement is more prevalent in those with SLE (5, 21). SLE is always a multiple-organ disease, so cardiac surgery is challenging in SLE patients. The kidney is frequently affected in SLE patients. Following major surgery among SLE patients, renal prognosis turns out poor and in-hospital mortality seems comparably high (22). Therefore, our study was initiated to explore prevalence, clinical contributors and subsequent outcomes of AKI following cardiac procedures in patients with SLE. More than half of the patients in our cohort developed postoperative AKI, indicating that AKI was a common complication after cardiac surgery in SLE patients. In the context of SLE pathologies, renal drug metabolism, together with anesthetic and surgical hits may accelerate the progression of kidney dysfunction. Moreover, increased valvular and vascular tissue fragility can be caused by SLE, as well as by long-term glucocorticoid use, increasing the difficulty of surgical suturing. Therefore, patients with SLE have longer surgery times, longer cardiac pulmonary bypass times, and greater risks of postoperative bleeding as well as blood transfusion (23–26). These factors might impair kidney function and make perioperative anesthesia management more challenging. Our results also revealed that AKI was associated with a worse prognosis, longer extubation time, longer ICU length of stay and more postoperative major complications. Clinicians must be watchful of AKI in SLE patients undergoing cardiac surgery.

Patients with eGFR less than 60 ml/min/1.73 m^2^ are considered to have moderate to severe renal dysfunction that may have clinical manifestations and significance (27). Moreover, it is eGFR, rather than the plasma creatinine concentration in conjunction with other factors, that is expected to be promising for enhancing discriminative ability of clinical predictive risk scores for AKI (28). Therefore, we chose 60 ml/min/1.73 m^2^ as the cutoff value when evaluating the relationship between preoperative renal dysfunction and postoperative AKI. eGFR has been associated with postoperative AKI in other studies. Gao and colleagues concluded that, of all incorporated factors in AKI prediction model, eGFR was one of the most important one (29). Takaki's confined in those undergoing off-pump coronary artery bypass grafting (30). Benaicha found the same in patients undergoing transcatheter aortic valve implantation (31). Our study further demonstrated that eGFR was associated with AKI in SLE patients, which is in accordance with the findings of the above studies, though those patients underwent cardiac surgery were not diagnosed with SLE. Low eGFR has been associated with chronic tubular dysfunction before surgery. Intraoperative hemodynamic changes, such as cardiac decompensation, as well as nephrotoxic drug administration, have been highlighted to further impair tubular function and thus renal dysfunction (30).

In our study, BMI, a continuous variable, was not significantly associated with AKI occurrence, but BMI ≥ 24 kg/m^2^ was. This peculiarity may have been due to the limited sample size. We chose 24 kg/m^2^ as the cutoff value because this value not only is the cutoff for overweight in our country but also enhances the likelihood of developing cardiac surgery–associated AKI in another study (32). Overweight patients have more metabolic comorbidities, such as hypertension, diabetes and hyperlipidemia, which result in a greater renal burden. The altered renal structure and hemodynamics in overweight patients may also increase kidney vulnerability to damage, increasing the likelihood of AKI after cardiac surgery (33, 34). Comprehensive preoperative evaluation, careful preoperative patient status optimization, strengthened intraoperative monitoring of hemodynamic and electrolyte status, use of goal-directed fluid therapy and transesophageal echocardiography, and higher intraoperative perfusion pressure control should be considered, especially in SLE patients with a preoperative eGFR less than 60 ml/min/1.73 m^2^ or a BMI greater than 24 kg/m^2^. Strengthened postoperative monitoring of renal function should also be taken into consideration for early detection of AKI.

Several limitations of our study should be acknowledged. First, the study was performed in a single center focusing on SLE patients receiving cardiac surgery. Our hospital is designated as a national referral center offering diagnostic and therapeutic care for complex and rare disorders. As far as we know, our cohort was the largest cohort uniquely incorporating SLE patients who experienced cardiac surgery. Even so, the sample size was still quite small due to the rarity of the disease. Therefore, these results should be interpreted carefully in other environments. Second, patients in our study were not evaluated on the SLE disease activity index (SLEDAI) before surgery, so SLE disease activity was not taken into consideration when evaluating the relationship with AKI. We instead chose inflammatory indices, including the erythrocyte sedimentation rate and C-reactive protein concentration, but these indices cannot fully reflect SLE disease activity. Nevertheless, most of our patients received elective surgery, so preoperative rheumatological evaluations were arranged for nearly every patient, and most of their SLE were considered to be in inactive or low disease activity state by physicians from the department of rheumatology and immunology. A matched comparison in patients with prevalent renal dysfunction but without SLE having the same type of cardiac surgery would be useful to examine prognosis and define possible modifying factors in SLE patients. Multicenter studies are needed to validate these findings.

Among SLE patients undergoing cardiac surgery, postoperative AKI prevails and is accompanied with poor prognosis. Patients with preoperative eGFR less than 60 ml/min/1.73 m^2^ or BMI over 24 kg/m^2^ should be given more attention because these factors seem to indicate a higher likelihood of developing postoperative AKI in SLE patients.

## Data Availability

The original contributions presented in the study are included in the article/Supplementary Material, further inquiries can be directed to the corresponding author.
